# Periocular motor neurotization: a systematic review of techniques and outcomes in orbicularis oculi reinnervation

**DOI:** 10.3389/fopht.2025.1687560

**Published:** 2025-11-24

**Authors:** Abdelrahman Abu Osba, Mohammad Abdullah, Oyeleye Oyesode, Ahsen Hussain

**Affiliations:** 1 Faculty of Medicine, Dalhousie University, Halifax, NS, Canada; 2 Department of Ophthalmology, Faculty of Medicine, Dalhousie University, Halifax, NS, Canada

**Keywords:** orbicularis oculi, blink restoration, nerve transfer, neurotization, muscle transposition, facial reinnervation, motor neurotization

## Abstract

**Background:**

Periocular motor neurotization is a surgical approach that can be indicated for the restoration of dynamic eyelid function in patients with facial nerve palsy. The orbicularis oculi muscle, responsible for eyelid closure, can be the target of a variety of re-innervation techniques, such as direct nerve transfers and muscle transpositions. This systematic review aims to map the existing evidence on direct neurotization techniques of the OOM, including nerve grafting, nerve transfers, and contralateral muscle transposition, and to describe their published effectiveness.

**Methods:**

A systematic search of PubMed, Embase, and supplemental searching with citation chaining using google scholar to identify studies detailing periocular neurotization techniques. Studies were included if they reported clear outcomes such as restored blinking or eye closure, improved corneal protection, and enhanced eyelid symmetry.

**Results:**

Of 857 screened studies, 6 met inclusion criteria, comprising 4 cohort studies, 1 case series, and 1 case report, with 106 patients eligible for detailed extraction. Direct neurotization of the OOM was associated with improvements in eye closure, blink reflex, and electromyographic data. Motor donor nerve selection was noted to affect outcomes significantly, with contralateral facial nerve branches yielding the highest blink improvement.

**Conclusions:**

This review highlights the emerging role of direct neurotization techniques in reinnervating the orbicularis oculi muscle for appropriate patients with facial nerve palsy. Future high-quality studies are needed to establish clear indications, long-term efficacy, and comparative advantages of direct OOM neurotization in clinical practice.

## 1 Introduction

Facial Nerve Palsy (FNP) is a significant event for patients with both functional and aesthetic consequences (1). The prognosis of the diagnosis depends primarily on its etiology and duration. The longer the paralysis persists and the more severe the levels of flaccidity, the less likely patients are to regain their baseline function or achieve aesthetic recovery. The orbicularis oculi muscle (OOM) is the primary muscle involved in the voluntary contraction of the eyelid and is responsible for eyelid closure, the blink reflex, and corneal protection (2). In facial palsy, OOM paralysis manifests as lagophthalmos, eyelid retraction and ectropion, which can lead to exposure keratopathy and, in more severe cases, corneal melt or perforation (3). On account of this, timely intervention to restore ocular protection is necessary. OOM reinnervation can be considered in appropriate cases to facilitate recovery and provide definitive ocular and visual rehabilitation. The timing at which reinnervation of the facial nerve is introduced following facial nerve injury is an important prognostic factor for functional recovery. Long-standing denervation with irreversible muscle atrophy will reduce the possibility of a meaningful return of function (4). Therefore, early intervention is often emphasized in treatment planning.

Historically, static eyelid support procedures, such as weight implants or tarsorrhaphy, have provided passive eyelid closure but cannot restore dynamic, spontaneous, or symmetrical movement (5). To address these limitations, dynamic reanimation techniques such as nerve transpositions, nerve grafts, and muscle transfers have been developed to reestablish neural input to the paralyzed facial musculature (6). One example is cross-facial nerve grafting, which involves harvesting a donor nerve and connecting a branch of the healthy facial nerve to the affected, paralyzed side. This method enables the transmission of facial nerve signals across the midline, restoring some degree of spontaneous and symmetrical movement (7). These indirect approaches aim to reestablish nerve function at the proximal branches of the facial nerve or through muscle transposition. However, they may not fully replicate the spontaneous activity of native facial musculature, which has a complex blink reflex and plays a crucial role in ocular protection. Unlike indirect methods that redirect neural input to the main facial nerve, direct neurotization establishes functional recovery by implanting donor nerve fascicles or transposing contralateral OOM fibers directly into the paralyzed OOM to restore physiological eyelid closure and reflexive blink mechanisms. Current direct neurotization strategies for the orbicularis oculi include nerve grafting, nerve transfers, and contralateral muscle transposition, each aiming to restore physiological eyelid function by targeting the native muscle directly.

Despite the increasing interest in periocular reanimation in patients with facial nerve palsy, there remains a lack of systematic organization of surgical techniques that directly reinnervate the orbicularis oculi muscle. Existing literature often combines direct and indirect reanimation techniques, making it difficult to assess specific outcomes and the clinical value of these methods. This systematic review aims to map the existing evidence on direct neurotization techniques of the OOM, including nerve grafting, nerve transfers, and contralateral muscle transposition, and to describe their published effectiveness. By understanding the proposed successful approaches and their application, this review will underscore the clinical relevance of direct motor neurotization (DMN) and provide a foundation for more targeted surgical planning.

## 2 Methods

Our study aimed to evaluate the effectiveness of direct neurotization techniques of the orbicularis oculi muscle as reported in the existing literature. We summarize the current evidence base of effective surgical approaches for direct neurotization of the OOM and identify the specific factors or conditions that contributed to their success.

### 2.1 Search strategy

We conducted a comprehensive review of scientific literature on surgical techniques for direct neurotization of the orbicularis oculi muscle. Direct neurotization was defined as any surgical method which introduced nervous innervation directly to the OOM. The search strategy included Embase, PubMed, Ovid MEDLINE, Cochrane Central Register of Controlled Trials (CENTRAL) and an in-depth Google Scholar query and citation chaining to capture relevant studies from additional databases. The search strategy was developed in consultation with a librarian and is provided in the **Supplementary Material, Appendix 1**. PRISMA-SR guidelines were followed, and a flow diagram is available in **Supplementary Material Appendix 2**.

### 2.2 Inclusion and exclusion criteria

Our inclusion criteria comprised patients with FNP who underwent periocular neurotization targeting the OOM through direct nerve transfers, contralateral muscle transpositions, nerve grafting, and techniques involving direct insertion of nerves or muscle grafts into the OOM via incision. Studies were included if they reported clear outcomes such as restored blinking or eye closure, improved corneal protection, and enhanced eyelid symmetry. We excluded studies unrelated to the orbicularis oculi muscle, those not focused on its reinnervation, static procedures lacking dynamic components, muscle grafts that did not involve the OOM, studies without identifiable outcomes, and non-English publications.

### 2.3 Quality assessment

The Joanna Briggs Institute Critical Appraisal tools were used to assess the quality of the included studies by two independent reviewers (AA & OO) (8). The scores of each study were not used to exclude any studies but rather as a discussion point within the article.

### 2.4 Data extraction and analysis

Published articles were imported into Covidence for thorough screening and full-text review by three independent researchers. Any conflicts during screening and data extraction were resolved through discussion or adjudicated by a third reviewer. Data extraction was performed using a structured Excel spreadsheet. A descriptive analysis was conducted to summarize the various surgical approaches and their effectiveness in achieving direct neurotization of the orbicularis oculi muscle. Findings were presented using summary tables and narrative descriptions.

## 3 Results

**Figure 1:**
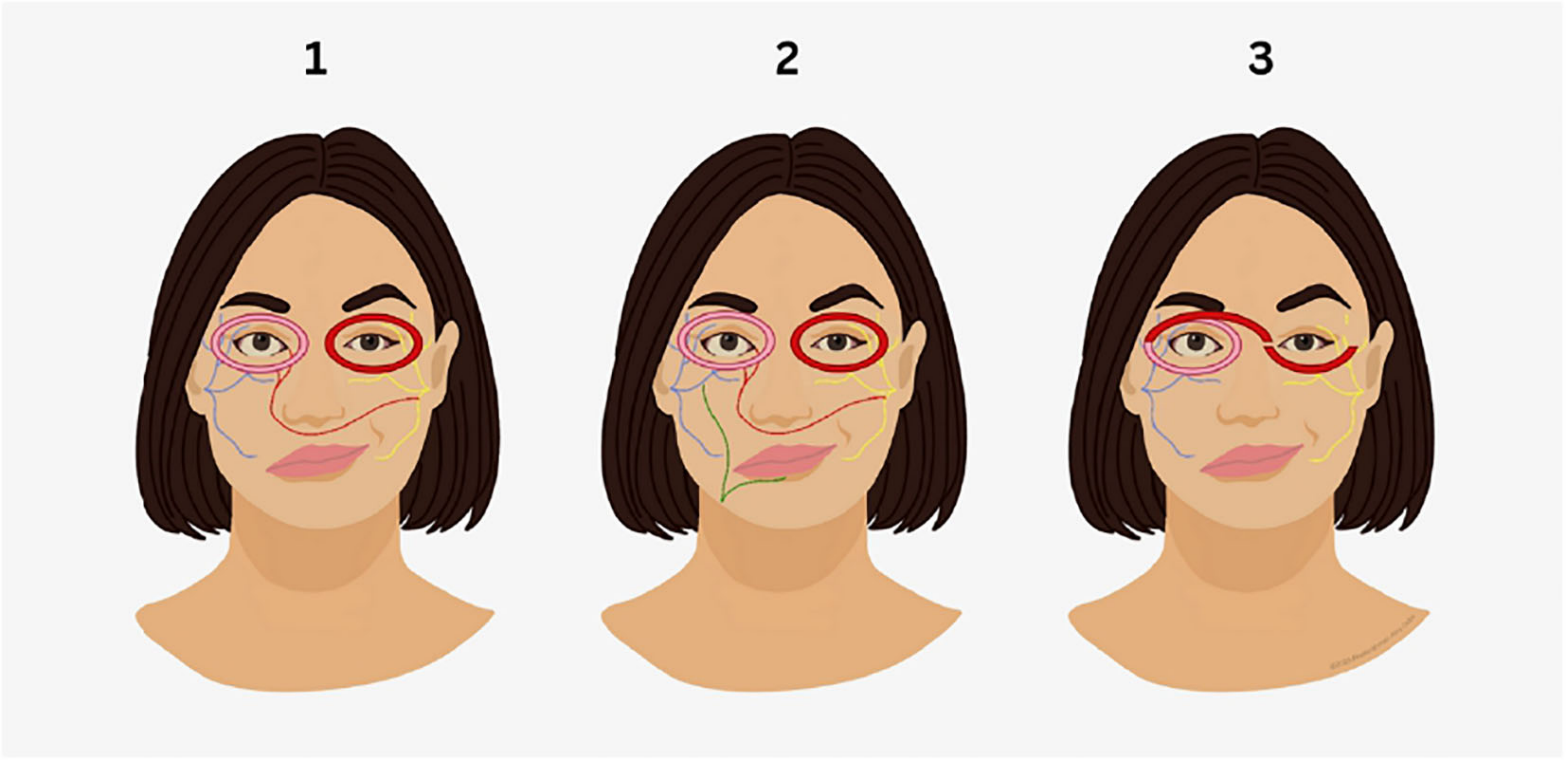
(Left) Direct neurotization: This technique involves the implantation of donor nerve fascicles directly into the paralyzed orbicularis oculi muscle. An interposition nerve graft (red) is proximally coapted to the motor donor nerve (yellow) and divided distally into fascicles that are implanted into both the upper and lower portions of the orbicularis oculi. (Middle) Direct neurotization combined with nerve transfer: In this approach, fascicles from the ipsilateral hypoglossal nerve (green) are redirected to the affected facial nerve (blue), providing additional motor input. An interposition graft is then used to deliver donor nerve fascicles (red) directly into the orbicularis oculi muscle, combining both nerve transfer and direct neurotization. (Right) Contralateral orbicularis oculi muscle transposition: A pedicled flap of the contralateral orbicularis oculi muscle, supplied by its native neurovascular pedicle (left eye), is tunneled across the nasal bridge and sutured to the paralyzed orbicularis oculi on the affected side (right eye).

Of the 857 study results retrieved, six studies were included in this systematic review, with publication years ranging from 2009 to 2021. The majority of studies were cohort (n = 4), with one case series (n = 1) and one case report (n = 1). As demonstrated in **Table 1**, overall methodological quality was acceptable. None of the included studies incorporated a true control group for comparison, and selection bias was a common concern due to the predominantly retrospective and non-randomized nature of the studies. The results were collated into three groups based on technique: Direct Neurotization (DN), Nerve Transfer (NT) combined with DN, and muscle transposition of the orbicularis oculi muscle, as illustrated schematically in **Figure 1**. Across the six studies, four examined DN only, two examined NT + DN, and two examined muscle transposition. A total of 323 patients were reported across all studies, with a mean age of 37.04 years (range: 4.3 to 78 years) and a mean postoperative follow-up duration of 17.5 months (6 to 44 months). The mean duration of facial palsy prior to intervention was 7.55 years (1 to 42 years). Of the 323 patients, 106 were eligible for detailed data extraction based on the predefined inclusion criteria; however, not all studies reported complete demographics, such as age, gender, or duration of paralysis. Reported outcome measures included eye closure, blink restoration, blink ratio, and electromyographic (EMG) improvement. Given the heterogeneity of study designs and outcome reporting, a quantitative synthesis was not feasible. In addition, as most studies comprised small retrospective series with moderate JBI scores, the reported improvements in blink function or EMG activity should be interpreted with caution.

**Table 1:** Critical appraisal of articles that met inclusion criteria.

Author, year by study type	Critical appraisal tool	Score
(Terzis & Karapydis, 2011) (9)	JBI Checklist for Cohort Studies	8/11
(Terzis & Karapydis, 2009) (10)	JBI Checklist for Cohort Studies	8/11
(Terzis & Karapydis, 2010a) (11)	JBI Checklist for Cohort Studies	8/11
(Terzis & Karapydis, 2010b) (12)	JBI Checklist for Cohort Studies	8/11
(Fernández-Pérez et al., 2021) (14)	JBI Checklist for Case Report	8/8
(Jamshidian-Tehrani et al., 2020) (15)	JBI Checklist for Case Series	8/10

### 3.1 Direct neurotization

Ninety-six patients across four studies (9–12) received direct neurotization of the orbicularis oculi muscle. Direct neurotization involved the implantation of donor nerve fascicles directly into the orbicularis oculi muscle. In the included studies, an interposition nerve graft was proximally coapted to the motor donor nerve and then distally divided into two segments, each further dissected into 2–3 fascicles for implantation into the upper and lower OOM, respectively. Clinical outcomes varied depending on motor donor selection, with the contralateral facial nerve demonstrating the highest blink improvement (15.25% - 43.8%). In comparison, ipsilateral cervical root (C) 7, C8, C12, and C4 & accessory nerve yielded moderate yet significant improvements (12.25% - 29.4%) (11, 12). Across these studies, eye closure and blink function were assessed using the grading criteria described by Terzis et. al, where a score of 1 indicates no eye closure/blink and a score of 5 indicates complete, eye closure/synchronous blinking (**Table 2**) (9–12). A significant improvement was found in eye closure and blink reflex, with postoperative scores improving from 2 to 3–4 across studies (9–11). Interestingly, younger patients (<7.5 years ± 3.2) exhibited greater blink improvement after direct neurotization in comparison to other dynamic reanimation procedures (12). Similarly, there were statistically significant improvements in EMG (1.21->2.1) as well as OOM function from 2 preoperatively to 3 postoperatively (9). In the 2009 study by Terzis and Karapydis (10), the mean improvement of eye closure and blink reflex was 36% and 34.25%, respectively. A summary of these outcomes, including blink ratio improvements, scoring data, and EMG findings, is presented in **Table 3**.

**Table 2:** Scoring system for eye closure and blink utilized by Terzis et al. (9, 11, 12) and retrieved from Terzis JK & Bruno W, (2002) (13).

Grading of eye closure			
Group	Grade	Designation	Grading of blink description
I	1	Poor	No eye closure (no contraction); maximal scleral show
II	2	Fair	Poor eye closure (min. contraction); 2/3 scleral show
III	3	Moderate	Incomplete eye closure; 1/3 scleral show
IV	4	Good	Nearly complete eye closure; minimal scleral show
V	5	Excellent	Complete eye closure; no scleral show

Grading of blink			
Group	Grade	Designation	Grading of blink description
I	1	Poor	No blink
II	2	Fair	Minimal blink (contraction)
III	3	Moderate	Initiation of blink present but only one-third amplitude
IV	4	Good	Some coordinated blink but only two-thirds amplitude
V	5	Excellent	Synchronous and complete blink present

**Table 3:** Improvement is reported as the mean difference between objective preoperative and postoperative scores, representing the percentage gain in function achieved using various direct neurotization techniques.

Authors	No. of patients	Blink ratio improvement based on motor donor (%)	Eye closure score (Pre → Post)	Blink score (Pre → Post)	EMG findings (Pre → Post)
(Terzis & Karapydis, 2011) (9)	26	N/A	2 → 3	2 → 3	1.21 → 2.1
(Terzis & Karapydis, 2009) (10)	23	N/A	2 → 3	2 → 3	1.12 → 2.31
(Terzis & Karapydis, 2010a) (11)	21	c. VII (c.OOM) – 15.25 c. VII (mandibular) –14.45 Ips. XII – 12.25	N/A	2 → 4	N/A
(Terzis & Karapydis, 20010b) (12)	26	c. VII (c.OOM) – 37.67 c. VII (zygomatic) – 43.8 Ips. XII – 28.7 Ips. C7 – 24.38 Ips. C8 – 17 Ips. C4 & XI – 29.4	N/A	N/A	N/A

Blink ratios were calculated based on donor-specific outcomes. EMG activity was recorded pre- and postoperatively, while changes in eye closure and blink reflex were assessed using the grading system shown in **Table 3**. c. VII (c.OOM): contralateral facial nerve branches supplying the orbicularis oculi muscle; c. VII (mandibular): contralateral facial nerve (mandibular branch); c. VII (zygomatic): contralateral facial nerve (zygomatic branch); Ips. XII: ipsilateral hypoglossal nerve; Ips. C7: ipsilateral seventh cervical root; Ips. C8: ipsilateral eighth cervical root; Ips. C4 & XI: ipsilateral fourth cervical root and accessory nerve.

### 3.2 Nerve transfer + direct neurotization

Two studies (11, 12) involving six patients described the combined technique of mini hypoglossal-facial nerve transfer (XII-VII) followed by direct neurotization of the OOM. These studies utilized an interposition nerve graft, which was divided distally into two branches to target both the upper and lower portions of the orbicularis oculi muscle. For direct neurotization using a graft, motor donor nerves included contralateral facial nerve branches. In cases involving mini hypoglossal nerve transfer, the ipsilateral hypoglossal nerve was used as the donor nerve, with its fascicles redirected to the affected facial nerve. While small sample sizes limit generalizability, this technique demonstrated moderate to significant improvements in blink function (**Table 4**). In the study by Terzis and Karapydis (11), the authors used contralateral XII and VII nerves and noted a 15.52% improvement in blink ratio (p = 0.049) and an increase in eye closure score from 1 to 4. A separate study by the same authors used a combination of ipsilateral XII and contralateral VII as donor nerves and demonstrated the highest improvement in blink ratio (44.67%) among all studies; however, this result was not statistically significant, possibly due to the small sample size (12).

**Table 4:** Reported outcomes of patients who underwent combined nerve transfer (NT) and direct neurotization of the orbicularis oculi muscle (OOM).

Authors	No. of patients	Blink ratio improvement based on motor donor (%)	Eye closure score (Pre → Post)
(Terzis & Karapydis, 2010a) (11)	5	c. XII + c. VII – 15.25	1 → 4
(Terzis & Karapydis, 2010b) (12)	1	Ips.XII + c. VII – 44.67	N/A

Blink ratios were calculated based on donor-specific outcomes. Changes in eye closure was assessed using the grading system shown in **Table 3**. c. VII (OOM): contralateral facial nerve branches supplying the orbicularis oculi muscle; c. XII: contralateral hypoglossal nerve; Ips. XII: ipsilateral hypoglossal nerve.

### 3.3 Orbicularis oculi muscle transposition

Two studies (14, 15) investigated the transposition of the orbicularis oculi muscle (OOM) from the contralateral (unaffected) side to the affected eyelid. Jamshidian-Tehrani et al. (15) presented a case series involving three patients who underwent OOM transfer from the upper eyelid of the unaffected side to the upper and lower eyelid of the affected side. The muscle flap was longitudinally divided into two segments to address upper and lower eyelid dysfunction. Postoperatively, the authors reported improvement in House-Brackmann (HB) scores and regained normal motor unit action potentials, which had been absent preoperatively. Improvement in visual acuity was also reported in two patients from 20/100 to 20/50 and from 20/100 to 20/60 (**Table 5**). A separate case report by Fernández-Pérez et al. (14) described a similar OOM transposition without dividing the muscle flap. Preoperatively, the patient presented with lagophthalmos measuring 2.5 mm with forced contraction and 5 mm with spontaneous blinking, both of which fully resolved postoperatively. Additionally, the patient’s HB score improved from grade IV to grade II with no donor site morbidity and reported subjective outcomes such as reduced tearing, improved blink synchrony, and the return of involuntary blinking.

**Table 5:** Individual patient data from two muscle transposition studies reporting preoperative and postoperative House-Brackmann scores.

Study	Patient #	Muscle divided	House-brackmann score (Pre → Post)	Visual acuity (Pre → Post)
(Fernández-Pérez et al., 2021) (14)	1	No	4 → 2	N/A
(Jamshidian-Tehrani et al., 2020) (15)	2	Yes	8 → 6	20/100 → 20/50
(Jamshidian-Tehrani et al., 2020) (15)	3	Yes	6.5 → 3	20/100 → 20/60
(Jamshidian-Tehrani et al., 2020) (15)	4	Yes	7.5 → 4.5	N/A

Each row corresponds to a single patient, muscle division status, visual acuity, and facial nerve function scores before and after surgery using the House-Brackmann grading system.

## 4 Discussion

Direct muscle neurotization has been explored for over a century, with early experimental and clinical studies demonstrating the feasibility of nerve implantation into denervated muscle tissue (16). Foundational work has also established the potential for functional reinnervation, later supported by histological and animal model studies that confirmed the formation of neuromuscular junctions and partial restoration of muscle force (9). Earlier studies have demonstrated that axonal regeneration from an innervated muscle to a denervated counterpart is possible using nerve grafts. In particular, Kermer et al. reported successful reinnervation of the orbicularis oris muscle via a muscle–nerve–muscle technique, with histological confirmation of axonal growth into the target muscle even after prolonged denervation periods (17). These findings have contributed to a growing interest in direct muscle neurotization as a means of restoring native muscle activity to achieve more natural and spontaneous facial function.

This systematic review identified and analyzed existing literature on direct reinnervation of the OOM in patients with facial nerve palsy, specifically through nerve grafts, nerve transfers, and muscle transpositions. Across the studies included in our review, direct neurotization of the OOM was associated with improvements in eye closure, blink reflex, and EMG data. It was also found that pediatric patients demonstrated greater blink improvement after DMN compared to other dynamic reanimation procedures (12). Motor donor nerve selection was noted to affect outcomes significantly, with contralateral facial nerve branches yielding the highest blink improvement. These findings emphasize the importance of motor donor nerve selection in facial reanimation, whilst also suggesting that direct targeting of the OOM may have unique advantages, including the potential restoration of the natural blink reflex and the minimization of synkinetic movements. However, the results were derived from single-center, non-comparative studies, which restrict generalizability and introduce potential bias. Direct neurotization of the orbicularis oculi muscle involves direct placement of donor nerves in the OOM through nerve grafts, nerve transfers, or contralateral muscle transposition to restore original function. A relatively newer technique of interest involves contralateral OOM muscle transposition, which has been anatomically validated in a cadaveric experimental study to allow the transfer of a muscle flap spanning over 50% of the contralateral upper eyelid length (18). The intrinsic facial nerve innervation is preserved in contralateral OOM muscle transposition, and reinnervation success can provide involuntary blinking directly on the side of the injury (14, 15). In our study, contralateral OOM transfers were associated with promising functional results, with no donor site morbidities reported at follow-ups ranging from 6 to 12 months. Patients also demonstrated preserved function of the contralateral (normal) eyelid, with no evidence of lagophthalmos or functional impairment (14, 15). However, lateral canthal tightening performed by Jamshidian-Tehrani et al. may have also contributed to improvements in eyelid closure and corneal protection, representing a combination of dynamic and static effects (15). Another technique includes direct neurotization using a nerve graft, wherein fascicles from a donor nerve are implanted directly into the orbicularis oculi muscle to restore voluntary contraction and dynamic eyelid closure (10). In comparison, nerve transfer involves leaving the donor nerve proximally intact while redirecting its distal end, or a portion of the nerve, to the target nerve, as seen in hypoglossal-to-facial nerve transfers. Studies by Terzis et al. have illustrated that the mini-hypoglossal approach, which employs partial nerve coaptation techniques, can preserve donor nerve function without incurring a significant impact on donor muscle function, such as tongue atrophy or speech and swallowing difficulties, which are typical downsides of the more traditional hypoglossal or accessory nerve transfers. Furthermore, end-to-side techniques using masseteric and accessory nerves have helped address donor site weakness, further demonstrating the safety profile of direct neurotization procedures (4) One of the main contraindications to direct neurotization is muscle atrophy due to prolonged denervation of the OOM. This can be suspected based on the clinical picture or assessed more objectively through EMG, particularly through the detection of fibrillation potentials. Patient age should also be considered as a relative contraindication to direct neurotization due to factors such as elevated oxidative stress and reduced neurotransmitter availability, which can contribute to poor reinnervation outcomes (19).

Several mechanisms have been proposed to explain the functional recovery observed after direct muscle neurotization. Increased Motor End Plates (MEPs) in the denervated muscle and the formation of new MEPs through the ‘Adoption Phenomenon’ were proposed. Another hypothesized mechanism involves axonal sprouting and direct ingrowth into denervated muscle fibers following nerve implantation (20). Corneal Neurotization is an example of a successful direct neurotization technique in regaining corneal sensation (21). Terzis et al. established this technique by using the contralateral supratrochlear or supraorbital nerves and tunneling them to the affected corneal limbus (21). This was followed by further modifications, including endoscopic and minimally invasive techniques, which are still being investigated (22).

Direct muscle neurotization is intended to provide an effective and relatively less invasive method to reinnervate the OOM and regain its functional capacity. However, the scarcity of case series data, the lack of standardized outcome measurements, and the limited surgical expertise for this novel approach make assessing its potential more challenging. This review found significant variation in outcome measures used to determine the success of direct neurotization. While some studies employed standardized, objective measures, such as the House-Brackmann grading scale or improvement in blink ratio in millimeters, many studies utilized author-developed measures, including the Terzis scoring system (9–12). Furthermore, only a few studies included electromyography assessments despite their role in confirming reinnervation. The variability in outcome measures poses a significant limitation when comparing the results across each study. Yao et al. highlighted similar limitations in the literature on measuring outcomes regarding reinnervation and blink reflex (23). For example, Jamshidian-Tehrani et al. did not look at spontaneous blinking or ocular surface conditions, nor did they differentiate between spontaneous voluntary closure, which was evident in pictures demonstrating Bells phenomenon (15). Additionally, Terzis and Karypidis included patients with prior static procedures, therefore adding a potential confounding variable in outcomes reporting (11). These factors demonstrate the importance of evaluating eyelid function, including clear documentation of spontaneous vs. voluntary closure, ocular surface conditions, and surgical history. In the study by Yao et al. (23), the authors advocate for the use of the Cornea, Static Asymmetry, Dynamic, Synkinesis (CADS) grading scale as a better way to characterize periocular outcomes in facial palsy as compared to general scales such as House-Brackmann or Sunnybrook; Yet this scale was not utilized in any DMN study included in our study. Therefore, there is a clear need to develop and validate standardized assessment methods for evaluating periocular reinnervation outcomes, allowing for meaningful comparisons across surgical interventions. Another significant limitation is the absence of granularity in demographic and clinical data specific to the direct neurotization cohorts. While many studies reported age, sex, etiology, and duration of palsy for the entire patient population, they rarely provided these specifics for the subset of patients who received direct neurotization of the OOM only. On account of this, it was not feasible to isolate these variables for analysis or to ascertain how they may have influenced this specific group’s outcomes. This lack of data makes it challenging to delineate precise clinical indications and to identify which patients would benefit most from DMN compared to other reanimation options. Finally, the findings of this review are derived from a limited evidence base, primarily small, single-center, non-comparative case series. Given the heterogeneity in patient selection, surgical techniques, outcome measures, and the lack of standardized reporting, the overall strength of evidence remains low and precludes definitive conclusions or meta-analytic synthesis. Despite these limitations, this review provides an essential foundation for understanding the emerging role of direct OOM neurotization techniques. Moving forward, more high-quality, prospective research employing uniform outcome measures are needed to explore these surgical techniques further and define the criteria for patient selection.

## 5 Conclusions

This systematic review highlights the emerging role of direct neurotization techniques in reinnervating the orbicularis oculi muscle for appropriate patients with facial nerve palsy. The findings suggest that direct approaches can yield meaningful functional outcomes while minimizing donor site morbidity, particularly when applied early and with appropriate motor donor selection. Future high-quality studies are needed to establish clear indications, long-term efficacy, and comparative advantages of direct OOM neurotization in clinical practice.

## Data Availability

The original contributions presented in the study are included in the article/**Supplementary Material**. Further inquiries can be directed to the corresponding author.
