# Supplementary figures and images for: Periocular motor neurotization: a systematic review of techniques and outcomes in orbicularis oculi reinnervation
Source: Front Ophthalmol (Lausanne). 2025 Nov 24;5:1687560. doi: 10.3389/fopht.2025.1687560 (PMC12682644; doi:10.3389/fopht.2025.1687560)

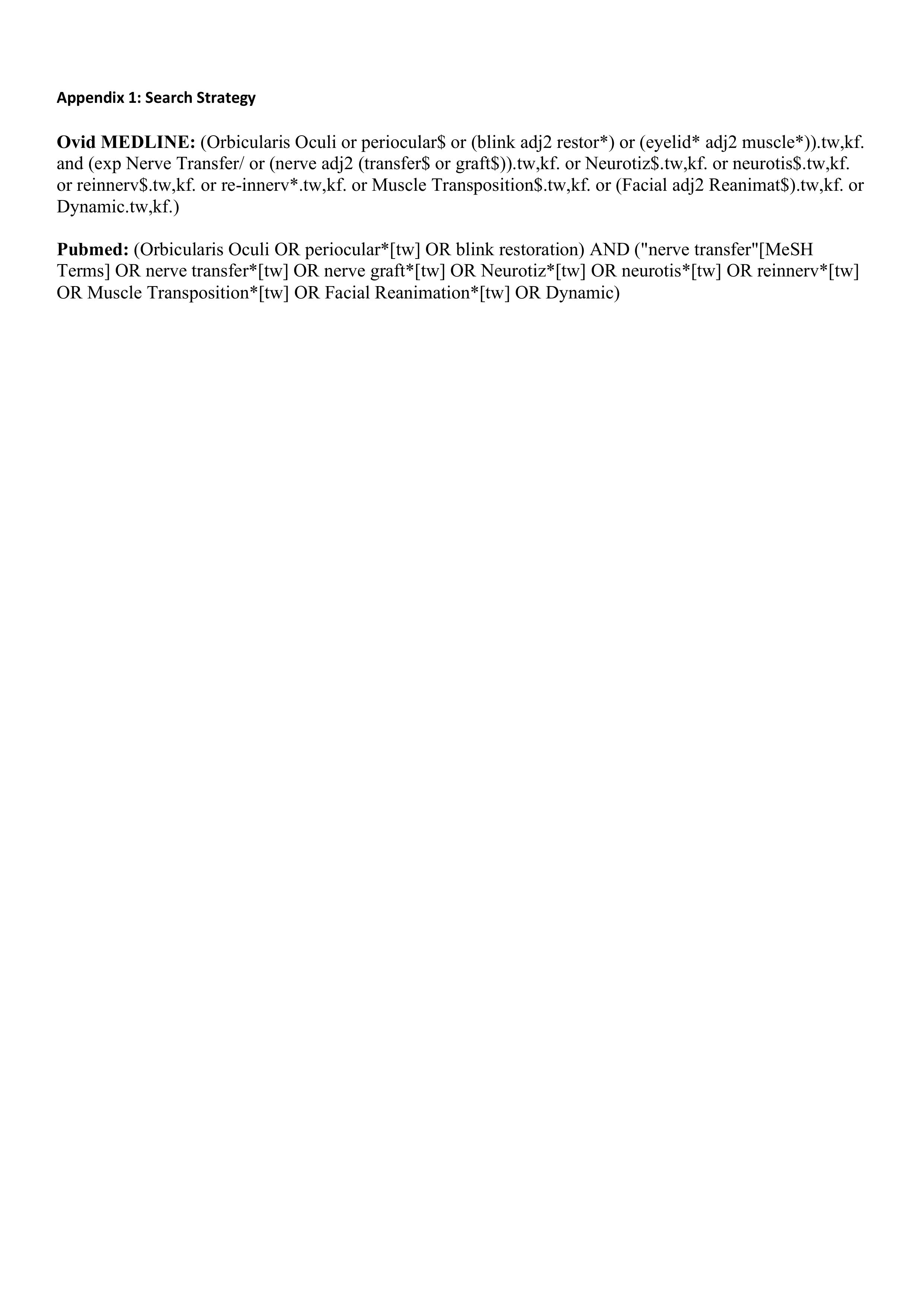

Supplement: Supplementary file 1 [file Image1.jpeg]

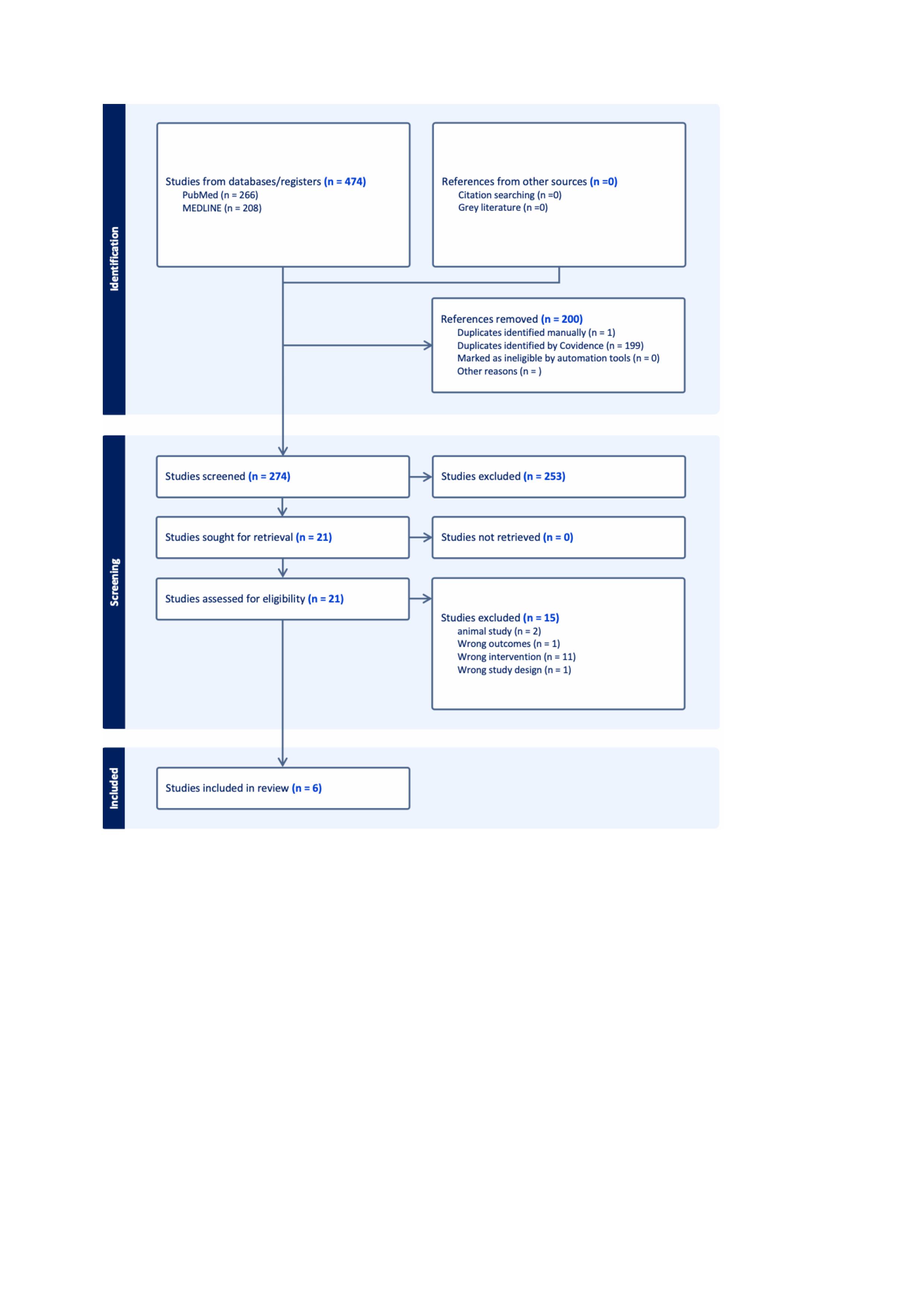

Supplement: Supplementary file 2 [file Image2.jpeg]

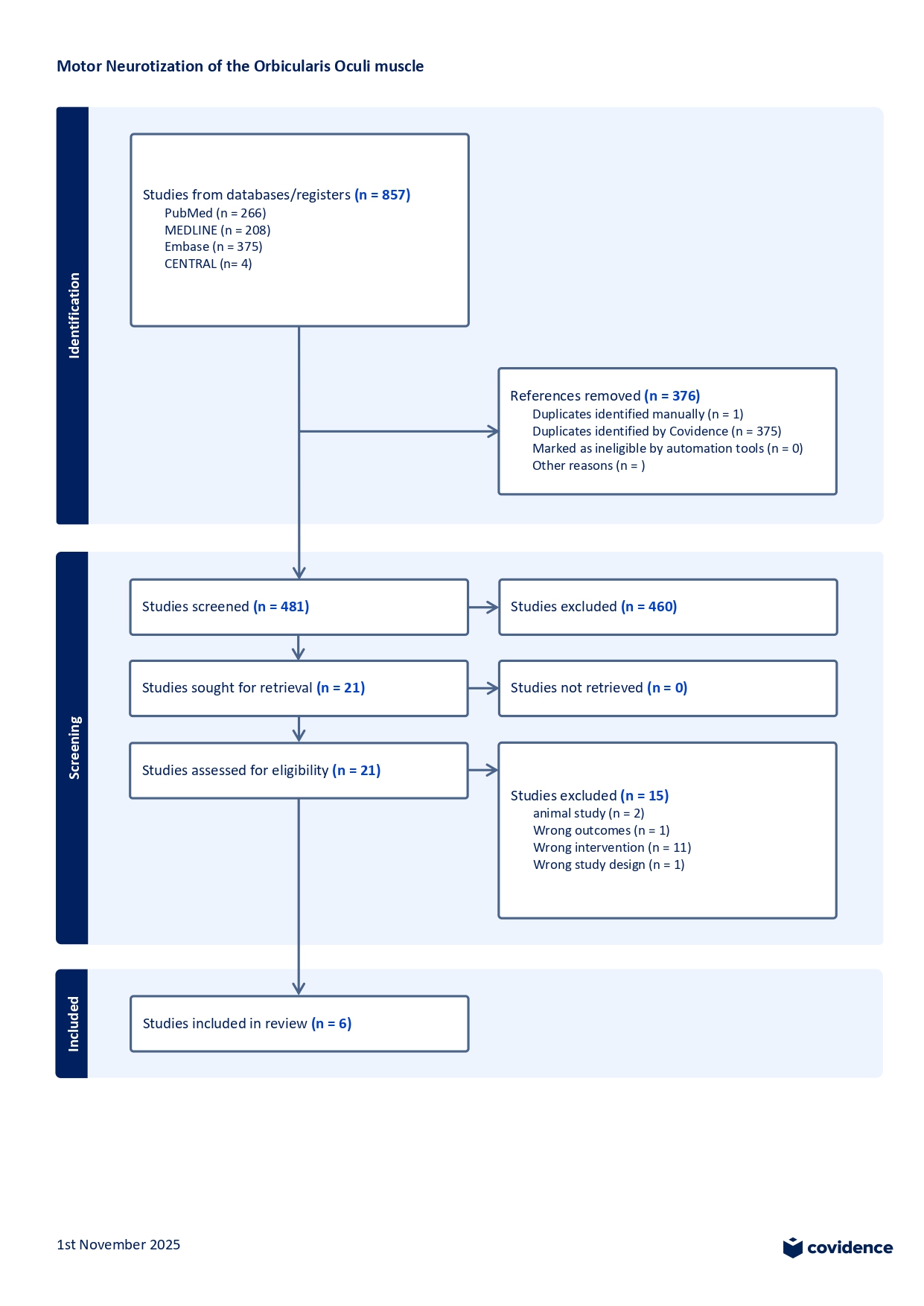

Supplement: Supplementary file 3 [file Image3.jpeg]

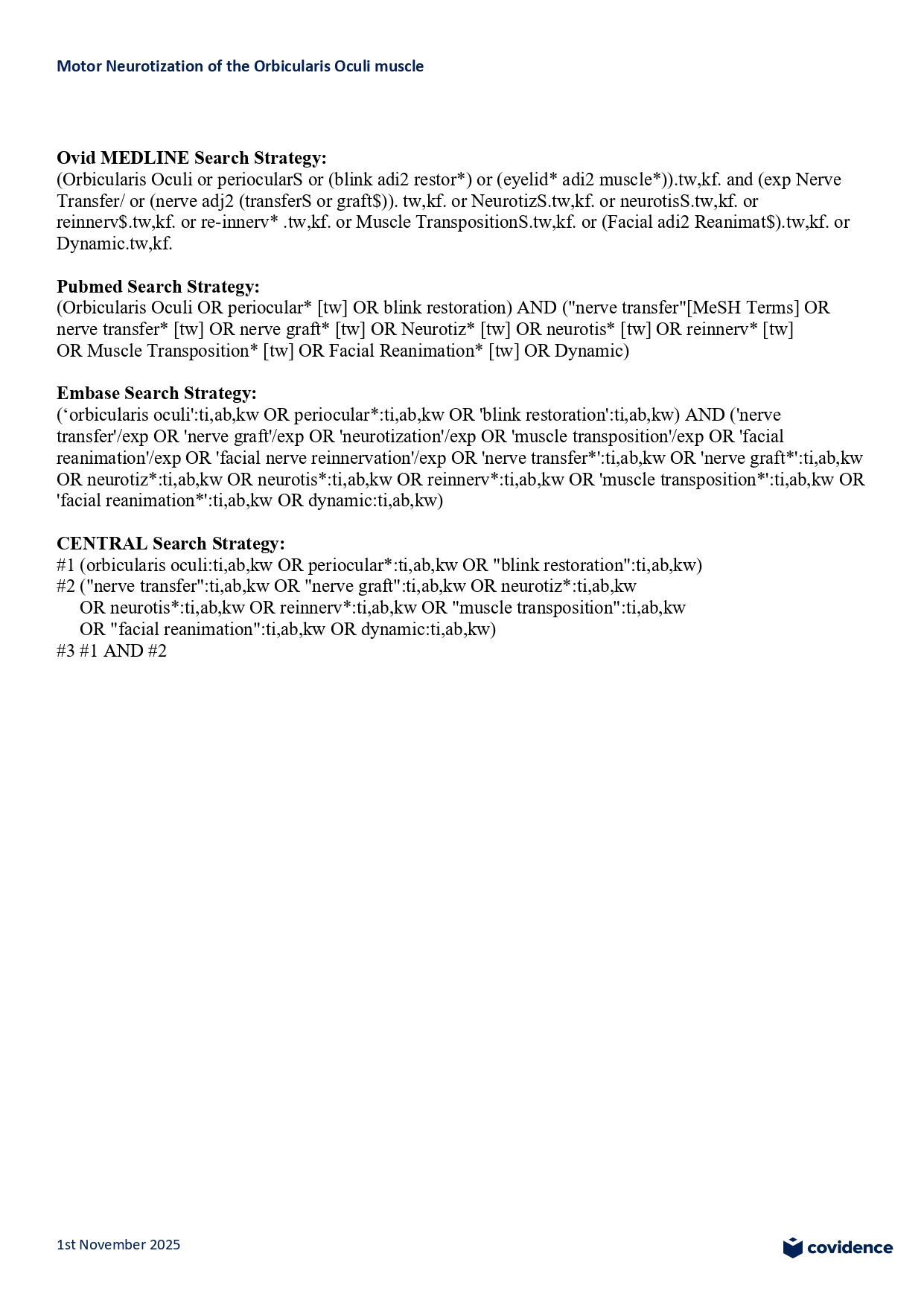

Supplement: Supplementary file 4 [file Image4.jpeg]
